# *Cited2* regulates proliferation and survival in young and old mouse cardiac stem cells

**DOI:** 10.1186/s12860-019-0207-2

**Published:** 2019-07-17

**Authors:** Qiong Wu, Qin Liu, Jinxi Zhan, Qian Wang, Daxiu Zhang, Shuangli He, Shiming Pu, Zuping Zhou

**Affiliations:** 1 School of Life Sciences, Guangxi Normal University, Guilin, 541004 China; 2 Guangxi Universities Key Laboratory of Stem cell and Biopharmaceutical Technology, Guangxi Normal University, Guilin, 541004 China; 3 Research Center for Biomedical Sciences, Guangxi Normal University, Guilin, 541004 China

**Keywords:** Cardiac stem cells, *Cited2*, Aging, Apoptosis, Proliferation

## Abstract

**Background:**

Cardiac stem cells (CSCs) exhibit age-dependent characteristics. *Cited2* has been implicated in the regulation of heart development; however, there is little known about how *Cited2* affects CSC aging.

**Results:**

*Cited2* mRNA and protein level was downregulated in aging heart tissue and CSCs. Old (O)-CSCs showed decreased differentiation and proliferation capacities as compared to Young (Y)-CSCs, the decrease in cell proliferation, increase in apoptosis, and cell cycle arrest in G0/G1 phase in CSCs are mediated by knocdown CITED2expression in (Y)-CSCs.

**Conclusions:**

*Cited2* plays an important role in cell cycle progression and in maintaining the balance between CSC proliferation and apoptosis in the process of aging without influencing cell fate decisions. These findings have important implications for cell-based therapies for heart repair.

**Electronic supplementary material:**

The online version of this article (10.1186/s12860-019-0207-2) contains supplementary material, which is available to authorized users.

## Background

Cyclic AMP response element binding protein(CREB) binding protein (CBP)/p300-interacting transactivator with ED-rich tail (CITED)2 is a transcriptional regulator that is critical for mice cardiac development and embryogenesis, loss of *Cited2* during gestation is lethal [1, 2]. *Cited2* deficiency in mice causes defects in arterial and ventricular septum and outflow tracts [1, 3, 4], while deletion of *Cited2* in mouse embryonic stem cells (ESCs) results in aberrant mitochondrial morphology, reduced glucose oxidation, increased glycolysis, and defective differentiation [5]. *Cited2* was identified for the maintenance of stem cell function, indicated to the maintenance of self-renewal of mouse embryonic stem cells through the regulation of Nanog expression [6], and was shown to regulate quiescence and apoptosis in adult hematopoietic stem cells [7] and regulate hematopoiesis in the fetal and adult liver [8]. *Cited2* was also found to play a critical role in cardiac development, the *Cited2*(−/−) mice developed laterality defects, including right isomerism, abnormal cardiac looping and hyposplenia [1, 2], and we have shown that *Cited2* expression was reduced in aged tendon-derived stem/progenitor cells, which may also influence TSPC differentiation [9].Other reports indicated that the downregulation of *Cited2* in aged TSPC contribute to TGFβ-mediated senescence [10]. We also reported significant changes in CSC stemness characteristics with advancing age [11]. However, whether *Cited2* controls CSC multipotency, differentiation, and aging remains unclear.

To address this issue, the present study investigated the role of *Cited2* in the maintenance of stemness and multipotency in aging murine CSCs using a gene targeting approach.

## Materials and methods

### Experimental animals and tissue collection

All procedures involving animals were approved by the Institutional Animal Care and Use Committee, Guangxi Normal University, Guilin, China. Young and old CSCs (Y- and CSCs, respectively) were prepared and cultured as previously described [11, 12].

### Microarray gene expression data

Gene expression data were obtained by microarray using the Affymetrix Mouse Genome 2.0 Array (Santa Clara, CA, USA). After quality assessment, three Y-CSC and three O-CSC samples were processed and normalized with the Robust Multichip Average [13] method. Details regarding the samples, mRNA processing and hybridization, and data access are described in our other papers [11, 12].

A detailed description of the gene expression analysis of C57BL/6 J mouse heart tissues with aging (2–3 months, 4 months, and 18 months; also based on the Affymetrix Mouse Genome 2.0 Array) and sample inclusion and exclusion criteria have been previously published [13, 14]. Corresponding CEL files are publicly available on the Gene Expression Omnibus database (accession numbers GSE43556, GSM1024592–94, and GSE7196).

In the present study, we used data from heart tissue samples at three ages (2–3, 4, and 18 months) and cardiac stem cell samples from mice at two ages (2–3 and 20–24 months). Raw data were subjected to quantile normalization and then analyzed in GeneSpring GX (zcomSilicon Genetics, Redwood City, CA, USA). Differentially expressed mRNAs were compared by analysis of variance and significance was evaluated with Fisher’s exact test, with a false discovery rate < 0.05 and fold change ≥1.5. To obtain gene expression profiles over the course of aging and identify the most probable clusters generating the observed aging series, the Series Test of Cluster (STC) algorithm was used [15] and STC-Gene Ontology (GO) analysis was performed. Data normalization and analyses were performed on the web (https://www.gcbi.com.cn/gclib/html/index).

### Reverse transcription (RT)-PCR and droplet digital (dd)-PCR

Total RNA was isolated from heart tissues and CSCs using TRIzol reagent (invitrogen) according to standard protocols and reversed transcribed using the OneStep RT-PCR kit. Heart tissue and CSC cDNA served as the template for RT-PCR and dd-PCR, respectively, which were performed using the primers listed in Table 1. When the number of CSCs was small, we used dd-PCR to measure *Cited2* expression. The reaction was carried out as previously described [16] in a 20-μl volume containing 10 μl of 2× EvaGreen Supermix (Bio-Rad, Hercules, CA, USA), 8 μl diluted cDNA, and primer sets. Reactions were run in triplicate. *Cited2* expression level was calculated relative to that of glyceraldehyde 3-phosphate dehydrogenase.

**Table 1:** Sequences of *Cited2*-shRNA and the primers

Primer	Forward primer 5′ → 3′	Reverse primer 5′ → 3′
*Cited2* (for dd PCR and RT-PCR)	AAATCGCAAAGACGGAAGGA	TGAACTGGGAGTTGTTAAACCT
*Cited2*-shRNA1	TGGCAGACCATATGATGGCCATGAA	
*Cited2*-shRNA2	CCCACAACCACTACATGCCGGATTT	
*Cited2*-shRNA3	GTCAGCTGTTGACTCGGTTAA	
*Cited2*-cDNA	tatcgaattc(EcoRI)ATGGCAGACCATATGATGGCCATGAAC	tatcggatcc(BamHI)ACAGCTGACTCTGCTGGGCTGCT
GAPDH	GCTGCCCAGAACATCATCCCT	TGAAGTCGCAGGAGACAACC

### Immunoblotting

The cells were prepared using RIPA buffer (Cell Signaling Technology) supplemented with 1 mM PMSF and phosphatase inhibitor cocktail (0.5 mM imidazole, 0.25 mM sodium fluoride, 0.3 mM sodium molybdate, 0.25 mM sodium orthovanadate, and 1.0 mM sodium tartrate). Protein concentrations were measured using a modified Lowry assay. 25–40 μg protein were separated by SDS-PAGE gel (10% acrylamide) and transferred onto PVDF membrane. Membranes were blocked in 5% non-fat dry milk (w/v) for 1 h then treated with primary antibody overnight at 4 °C. The antibody GAPDH (1:1000) and IgG (1:10000) from Cell Signaling Technology, antibody CITED2from Abcam (1:1000).

### Lentivirus construction and infection

For knockdown experiments, oligonucleotides targeting *Cited2* were used to clone short hairpin (sh)RNA-encoding sequences into the hU6-MCS-PGK-EGFP lentiviral RNAi vector (Hanbio, Shanghai, China). To generate the overexpression construct, *Cited2* cDNA cloned by PCR using the primers listed in Table 1 was inserted into the CMV-MCS-IRES-EGFP lentiviral vector (Hanbio). Recombinant knockdown and overexpression lentiviral vectors were produced by co-transfection of 293 T cells with the pSPAX2, pMD2G, and pHBLV plasmids using LipoFiter reagent (Hanbio). Lentivirus-containing supernatant was collected 48 h after transfection and passed through a 0.45-μm cellulose acetate filter (Millipore, Billerica, MA, USA). Recombinant lentiviruses were concentrated by ultracentrifugation for 2 h at 72,000×*g*.

For lentiviral infection, CSCs were cultured until they reached ~70% confluence, and the culture medium was removed before infection. The cells were washed with phosphate-buffered saline and treated with the virus-containing medium (multiplicity of infection ~ 50, combined with 5 μg/ml polybrene). After 24 h, the culture medium was replaced with fresh medium. Most of the CSCs (> 80%) expressed enhanced green fluorescent protein (EGFP) 72 h after transduction. The empty vector encoding EGFP was used as a negative control. CSCs were harvested 3 days after viral infection and *Cited2* expression level was evaluated with dd-PCR.

### Stem cell differentiation and immunofluorescence analysis

After 3 days viral infection, lentivirus-infected and negative control CSCs were cultured in cardiomyocyte, smooth muscle cell, and endothelial differentiation induction medium for 14 days, then examined for the expression of cardiac lineage-specific markers by immunocytochemistry as previously described [11, 12].

### Analysis of cell cycle, proliferation, and apoptosis

Y−/O-CSCs were transducted with *Cited2*-shRNA, *Cited2-*cDNA, and negative control lentiviral particles; 48 h later, cell apoptosis was detected by flow cytometry using an Annexin V-phycoerythrin (PE) Apoptosis Detection kit (Invitrogen, Carlsbad, CA, USA). The percentage of apoptotic cells was counted as the sum of annexin V-PE single-positive and annexin V-PE/7-aminoactinomycin D (7-AAD) double-positive cells. The 5-ethynyl-2′-deoxyuridine (EdU) assay was performed to assess cell proliferation 48 h after lentiviral infection by incubating the cells in 10 μmol/l EdU solution (RiboBio, Guangzhou, China) for 2 h followed by flow cytometry analysis. Cells cycle analysis was performed with the 7-AAD Flow Cytometry Assay kit (Ebioscience, San Diego, CA, USA) according to the manufacturer’s instructions. The methods have been described in detail elsewhere [11, 12].

## Results

### Cited2 mRNA expression during heart and CSC aging

We compared mRNA expression profiles in heart tissue at 2–3, 4, and 18 months. A total of 5506 differentially expressed genes were identified by pairwise comparisons among groups, with a false discovery rate < 0.05 and fold change ≥1.5 in at least one of the pairwise comparisons. Hierarchical clustering of genes that were differentially expressed during heart tissue aging was performed (Additional file 1: Figure S1). STC and STC-GO analyses of the most probable set of clusters generating the observed aging series revealed 16 clusters of differentially expressed genes (Additional file 1: Figure S2) with six significant cluster profiles (profiles 14, 15, 10, 11, 9, and 1); a striking observation was made in three significant cluster profiles (profiles 11, 9, and 1). Considering that the gap in age between 2 and 3 and 4 months is small, we assumed that the gene expression profiles between these two groups would be similar, and therefore focused on cluster profiles 14 and 15 (Additional file 1: Figure S3A). Genes that were downregulated with heart aging in profile 15 (0, 0, − 1) included those related to transport, regulation of transcription, and cell differentiation (Additional file 1: Figure S3-B). The differentially expressed genes with aging in STC profile15 were listed in Additional file 2 and *Cited2* expression level was similar between 2 and 3 and 4 months mice, downregulation in 18 month mice heart (Additional file 2: Table S1).

The microarray analysis revealed that *Cited2* expression was downregulated during aging in heart tissue and cardiac stem cells (CSCs) and may play as a core senescence and regulator in CSCs.

### Cited2 is downregulated in O-CSCs as compared to Y-CSCs

**Fig. 1:**
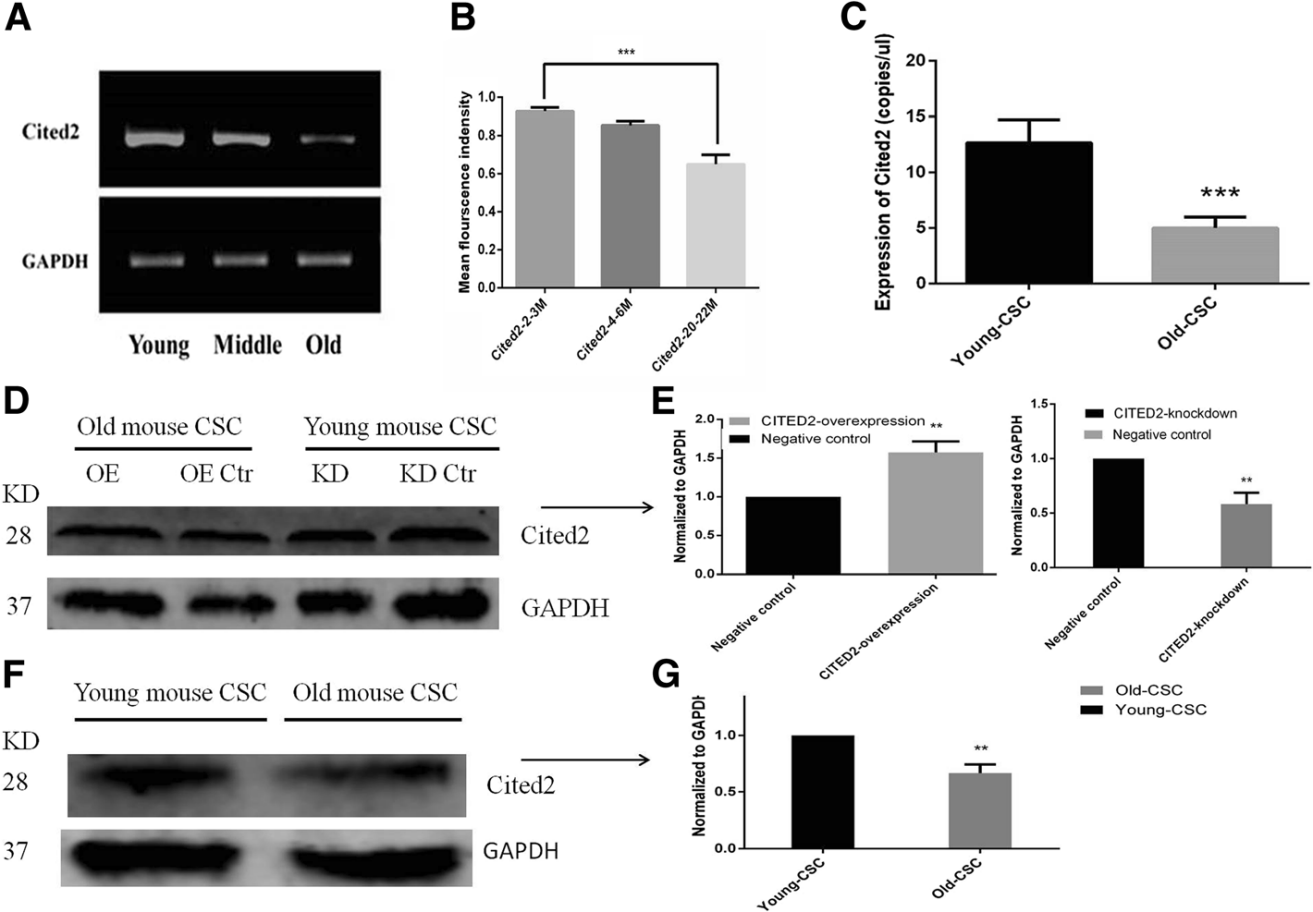
Age-dependent expression of *Cited 2* in mice heart tissues and CSCs. **a**. RT-PCR for *Cited2* mRNA expression in Young (2–3 months), Middle (4–6 months) and Old (20–22 months) mice heart tissues. **b**. The analysis of RT-PCR for *Cited2* mRNA expression. **c**. DD-PCR for *Cited2* mRNA expression in Young (2–3 months) and Old (20–22 months) CSCs. **d**-**g**. Immunoblotting of CITED2 protein levels in lentivector transduction groups and CSCs groups. OE represented Old CSCs transducted with lentiviral particles of *Cited2*-cDNA, KD represented Young CSCs transducted with lentiviral particles of *Cited2*-shRNA3, the control was transducted with empty lentivector. Data were from three independent experiments and presented as mean ± SD. Statistical analysis was implemented by GraphPad Prism 5, *p < 0.05, ** *p* < 0.01, and *** *p* < 0.001, Old-CSCs contrast to Young-CSCs

We examined *Cited2* gene expression level in 2–3, 4–6, and 20–22 month mouse heart tissues by RT-PCR and found that *Cited2* level decreased with aging (Fig. 1a, b). *Cited2* mRNA expression was also lower in O-CSCs than in Y-CSCs (*P* < 0.01; Fig. 1c).The CITED2 protein level was confirmed by western blotting, CITED2 protein was also lower in O-CSCs than in Y-CSCs (*P* < 0.01; Fig. 1f, g).

### The differentiation characteristics of O-CSCs with CITED2overexpression

**Fig. 2:**
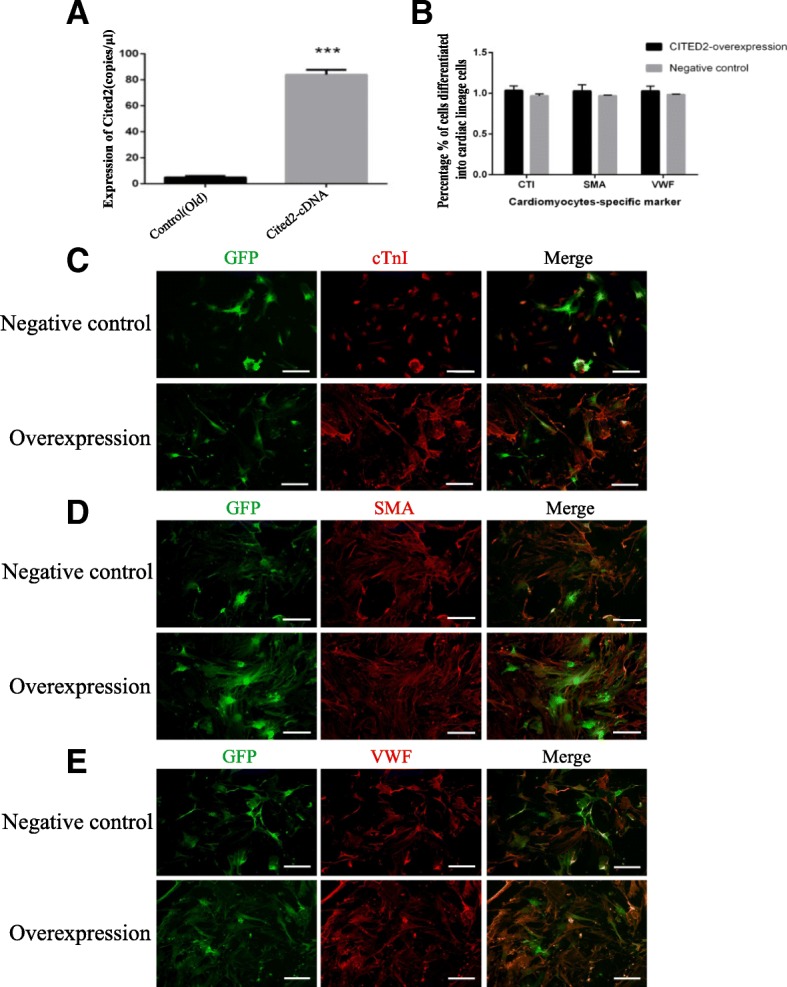
The affect of *Cited2* overexpression on differentiation in Old cardiac stem cells. **a**:DD-PCR of *Cited2* mRNA expression for lentivector transduction. The control was transducted with empty lentivector. Overexpression represented Old CSCs transducted with lentiviral particles of *Cited2*-cDNA; Data were from three independent experiments and presented as mean ± SD. Statistical analysis was implemented by GraphPad Prism 5, *p < 0.05, ** *p* < 0.01, and *** *p* < 0.001. **b**: The differentiation efficiency of CSCs from *Cited2* overexpression and negative control group. The percentage % of cells differentiated into cardiomyocytes was calculated cardiomyocytes marker cells among total GFP cells based on a mean of five independent experiments. Statistical analysis was implemented by GraphPad Prism 5, *p < 0.05, ** *p* < 0.01, and *** *p* < 0.001, contrast to control. **c**-**e**: Immunofluorescence staining (red) of cardiomyocytes-specific marker-cTnI; smooth muscle marker-α-SMA and enodthelial cell marker-VWF; transduction CSCs [17] staining with GFP; then merged images. Overexpression represented Old CSCs transfected with lentiviral particles of *Cited2*-cDNA; The control was transducted with empty lentivector. GFP-green is represented lentivirus vector. Scale bars, 50 μm. Each experiment is repeated five times, and the picture represents a single result

To examine the role of *Cited2* in aging mouse CSCs, we used a lentiviral vector to stably express *Cited2* in O- and Y-CSCs, with cells transducted with empty vector serving as a negative control. Transduction with the *Cited2*-cDNA vector for 48 h increased *Cited2* mRNA and protein levels in O-CSCs as compared to the negative control group (Figs. 1d, e, 2a). We then assessed the potential of O-CSCs to differentiate into cardiomyocyte, smooth muscle, and endothelial lineages upon transduction with *Cited2*-cDNA or control vector. After 14 days of incubation in the induction medium, lineage-specific markers were analyzed with immunocytochemistry. We have quantified the number of cells differentiated Markers and GFP, calculated the percentage % of cells differentiated into cardiac lineage cells, over the cells expressing GFP. Cells positive for cardiac troponin (cTn)I, α-smooth muscle actin (α-SMA), and Von Willebrand factor (VWF) were detected in both groups, with no difference in expression levels between them (Fig. 2b). The morphology of the differentiated cells were not observed (Fig. 2c-e). These results suggest that *Cited2* has no effect on the differentiation of O-CSCs.

### The differentiation characteristics of Y-CSCs with Cited2 knockdown

**Fig. 3:**
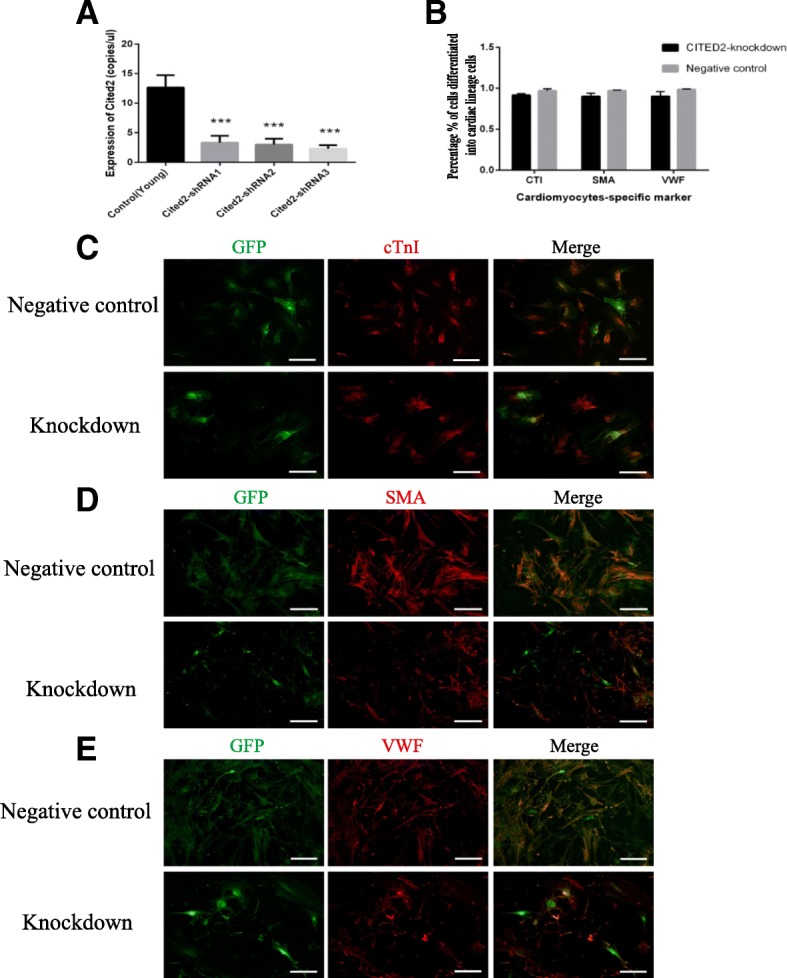
The affect of *Cited2* knockdown on differentiation in Young cardiac stem cells. **a**: DD-PCR of *Cited2* mRNA expression for lentivector transduction. The control was transducted with empty lentivector. Knockdown represented Young CSCs transducted with lentiviral particles of *Cited2*-shRNA1, *Cited2*-shRNA2 and *Cited2*-shRNA3; Data were from three independent experiments and presented as mean ± SD.Statistical analysis was implemented by GraphPad Prism 5, *p < 0.05, ** *p* < 0.01, and *** *p* < 0.001, contrast to control. **b**: The differentiation efficiency of CSCs from *Cited2* knockdown and negative control group. The percentage % of cells differentiated into cardiomyocytes was calculated cardiomyocytes marker cells among total GFP cells based on a mean of five independent experiments. Statistical analysis was implemented by GraphPad Prism 5, *p < 0.05, ** *p* < 0.01, and *** *p* < 0.001, contrast to control. **c-e**: Immunofluorescence staining (red) of cardiomyocytes-specific marker-cTnI; smooth muscle marker-α-SMA and enodthelial cell marker-VWF; transfection CSCs staining with GFP; then merged images. Each experiment is repeated five times, and the picture represents a single result. Knockdown represented Young CSCs transfected with lentiviral particles of *Cited2*-shRNA3; The control was transducted with empty lentivector. GFP-green is represented lentivirus vector. Scale bars, 50 μm

To further investigate the role of *Cited2* in mouse CSC aging, we knocked down *Cited2* expression in Y-CSCs using three different shRNAs (sequences are listed in Table 1). All three shRNA vectors decreased *Cited2* mRNA level 48 h after transduction (Fig. 3a). Y-CSCs transducted with empty vector served as a negative control. The CITED2 protein level was confirmed by western blotting, CITED2 protein was also lower in lentivirus *Cited2* -shRNA3-transduced Y-CSCs (*P* < 0.01; Fig. 1d, e). We then evaluated the potential for differentiation into cardiomyocyte, smooth muscle, and endothelial lineages in lentivirus *Cited2*-shRNA3-transduced Y-CSCs. After 14 days of incubation in the induction medium, lineage-specific markers were analyzed by immunocytochemistry. Cells positive for cTnI, α-SMA, and VWF were detected in both *Cited2* knockdown and control groups in similar numbers (Fig. 3b), indicating that *Cited2* does not influence the differentiation potential of Y-CSCs.

### Cited2 is required for CSC proliferation and survival

**Fig. 4:**
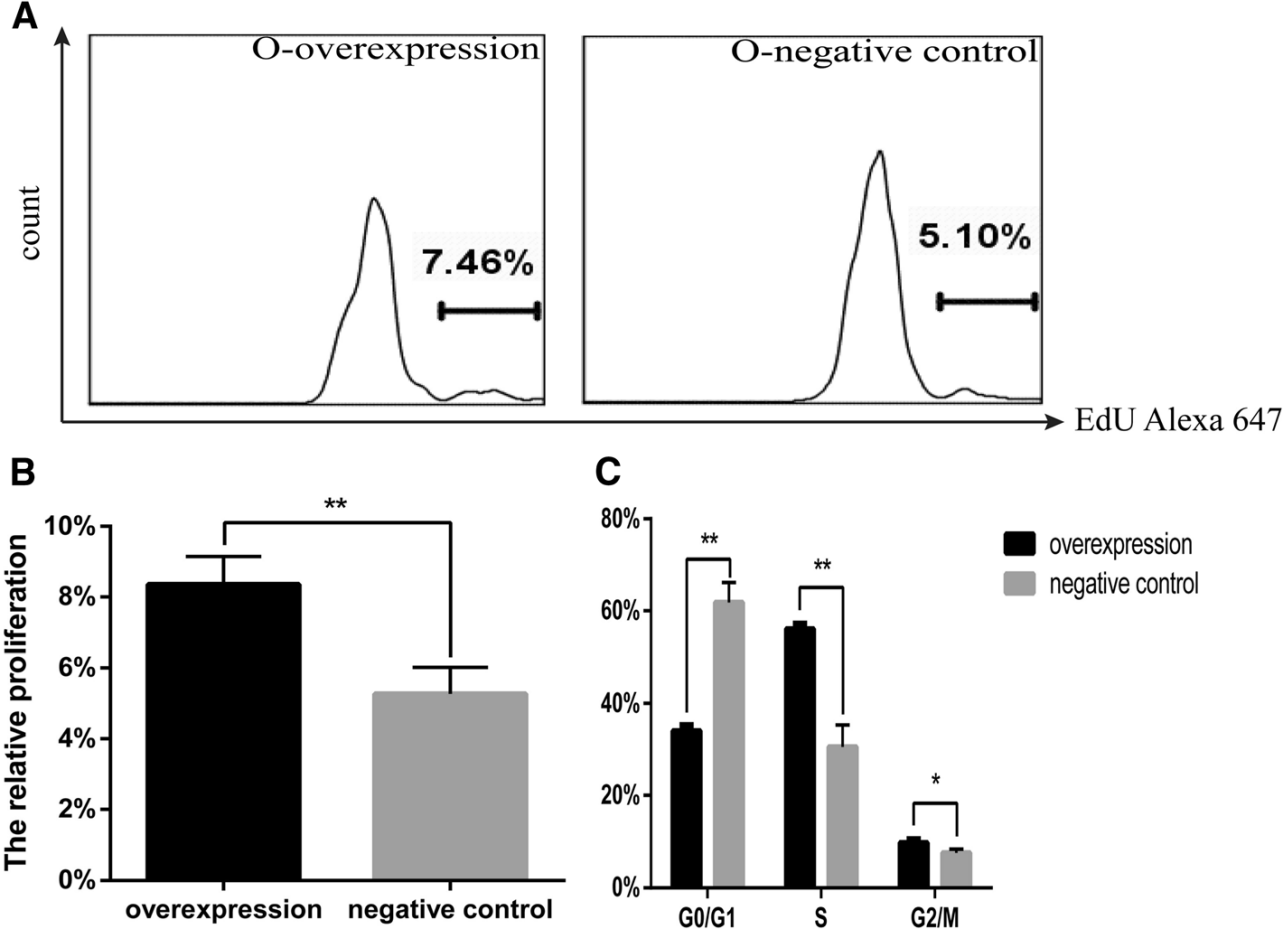
*Cited2* overexpression affects cell proliferation in Old CSCs. **a** The proliferation was tested by FCM with EdU assays. O-overexpression represented Old CSCs transducted with lentiviral particles of *Cited2*-cDNA. The control was transducted with empty lentivector. **b** Statistical analysis of proliferation rate was implemented by GraphPad Prism 5. **c** Cell cycle analysis was carried out by FCM, statistical analysis of apoptosis rate was implemented by GraphPad Prism 5, *p < 0.05, ** *p* < 0.01, and *** *p* < 0.001, contrast to control. Data are based on three independent experiments, and the picture represents a single result

*Cited2* was previously found to play a role in cell proliferation [18]. In order to verify these findings, we transducted O-CSCs with lentivirus overexpressing *Cited2* and then analyzed cell proliferation with the EdU assay. Upregulation of *Cited2* in O-CSCs increased the proliferative fraction relative to the control group (Fig. 4a, b). To confirm that *Cited2* promotes cell growth, we also evaluated the affects of *Cited2* on cell cycle distribution by flow cytometry. *Cited2* overexpression in O-CSCs reduced the G0/G1 fraction and increased the proportion of cells in S and G2/M phases of the cell cycle relative to the control group (Fig. 4c).

**Fig. 5:**
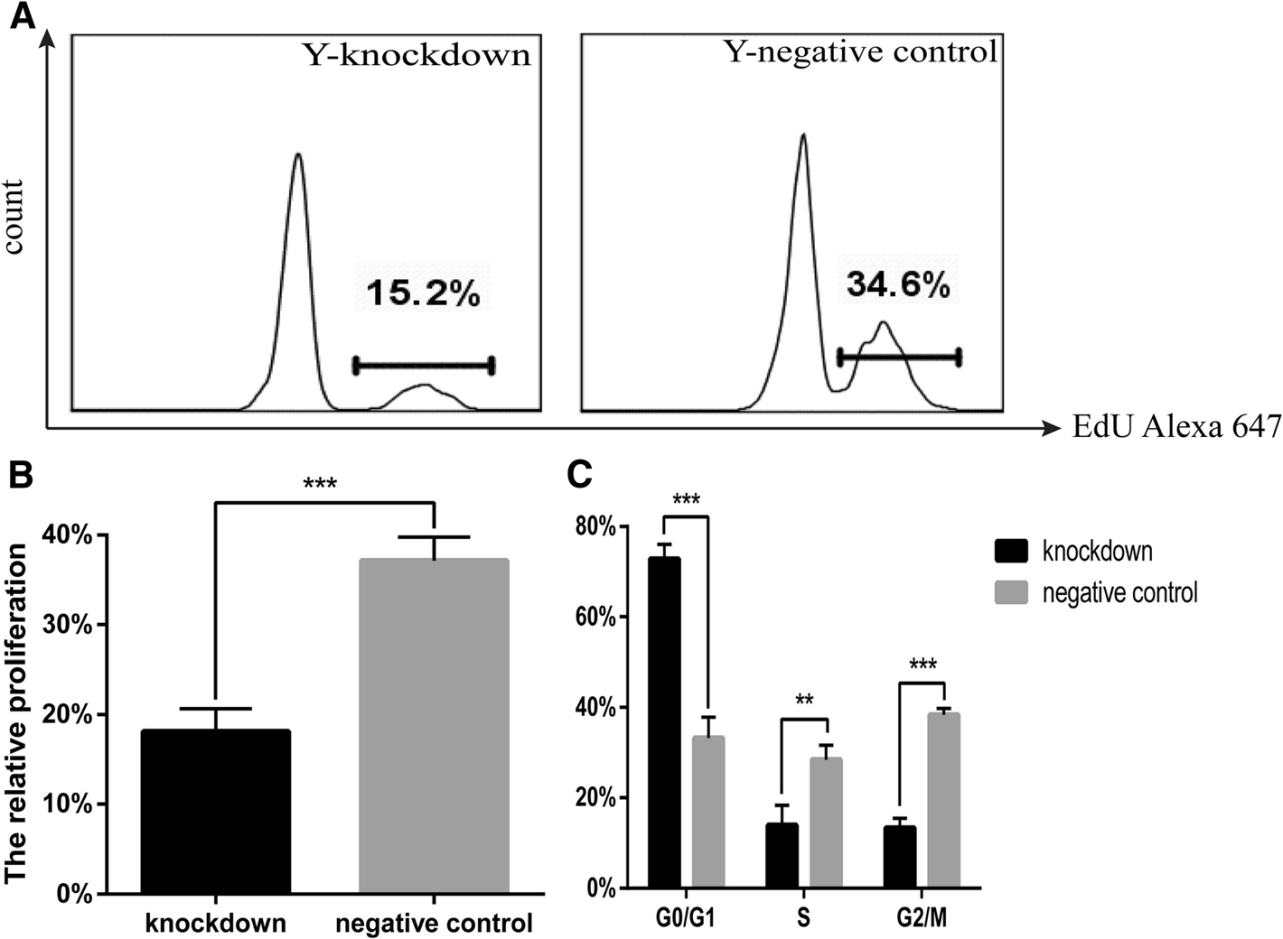
*Cited2* knockdown affects cell proliferation in Young CSCs. **a** The proliferation was tested by FCM with EdU assays. Y-knockdown represented Young CSCs transducted with lentiviral particles of *Cited2*-shRNA3; The control was transducted with empty lentivector. **b** Statistical analysis of proliferation rate was implemented by GraphPad Prism 5. **c** Cell cycle analysis was carried out by FCM, statistical analysis of apoptosis rate was implemented by GraphPad Prism 5, *p < 0.05, ** *p* < 0.01, and *** *p* < 0.001, contrast to control. Data are based on three independent experiments, and the picture represents a single result. Knockdown represented Young CSCs transducted with lentiviral particles of *Cited2*-shRNA3. The negative control was transducted with empty lentivector

To clarify the role of *Cited2* in mouse CSC proliferation, we transfected Y-CSCs with *Cited2*-shRNA vector and then evaluated EdU incorporation. *Cited2* knockdown in Y-CSCs reduced cell proliferation as compared to control cells (Fig. 5a, b). To confirm that *Cited2* promotes cell growth, the affects of *Cited2* silencing on cell cycle distribution was evaluated by flow cytometry. Cell cycle analyses revealed that downregulation of *Cited2* blocked Y-CSCs in G0/G1 phase and consequently reduced the sizes of the S- and G2/M-phase fractions relative to the control group (Fig. 5c).

**Fig. 6:**
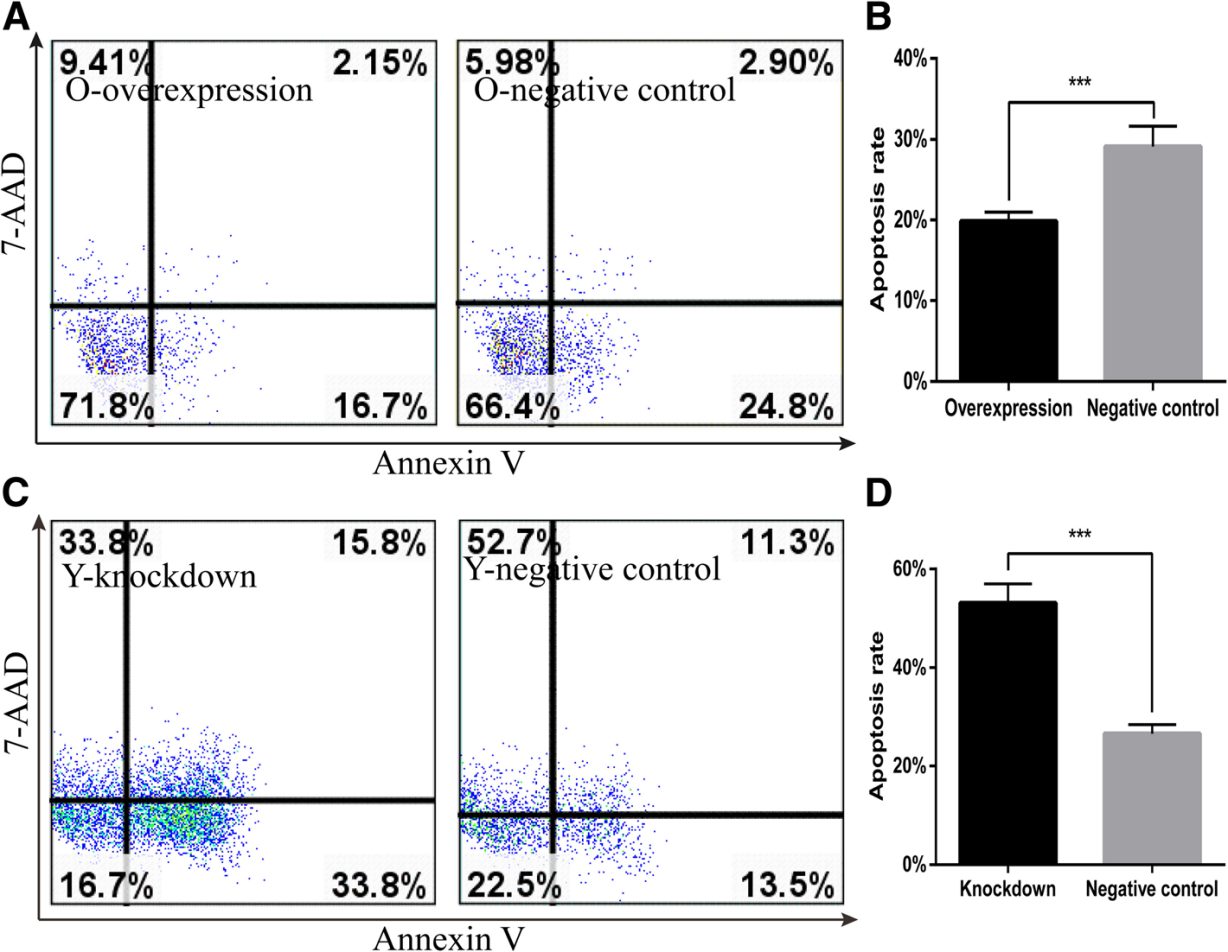
*Cited2* affects cell apoptosis with CSCs aging. The apoptosis was tested by FCM with Annexin V-PE apoptosis detection kit and the PE-positive cells percentage stand for the ratio of apoptosis cells to all numbered cells. **a** The apoptosis rate in Old CSCs transducted with lentiviral particles of *Cited2*-cDNA (left panel) and empty lentivector (right panel). **b** Statistical analysis of apoptosis rate was implemented by GraphPad Prism 5. **c** The apoptosis rate in Young CSCs transducted with lentiviral particles of *Cited2*-shRNA3 (left panel) and empty lentivector(right panel). **d** Statistical analysis of apoptosis rate was implemented by GraphPad Prism 5. *p < 0.05, ** *p* < 0.01, and *** *p* < 0.001, contrast to control. Data are based on three independent experiments and the picture represents a single result. Overexpression represented Old CSCs transducted with lentiviral particles of *Cited2*-cDNA. Knockdown represented Young CSCs transducted with lentiviral particles of *Cited2*-shRNA3.The negative control was transducted with empty lentivector

To gain further insight into the role of *Cited2* in mouse CSC aging, we analyzed apoptosis in Y-CSCs transfected with vectors encoding *Cited2* cDNA or shRNA. *Cited2* overexpression inhibited apoptosis as compared to control- transducted cells (Fig. 6a, b), whereas *Cited2* knockdown had the opposite effect (Fig. 6c, d). These results demonstrate that *Cited2* promotes proliferation while inhibiting apoptosis in CSCs.

## Discussion

Stem cell aging is critically important for organismal development and senescence. The therapeutic potential of CSCs is limited by the decline in their regenerative potential with donor age [11]. The molecular mechanism for stem cell regulation during aging is not well understood. *Cited2*, a transcription factor that is involved in the control of differentiation and self-renewal in several types of adult stem cell, is downregulated with advancing age [10]. Our results confirmed that *Cited2* is downregulated in the heart and CSCs of aging mice. A bioinformatics analysis indicated that changes in *Cited2* expression are associated intracellular transport, transcriptional regulation, cell differentiation, and heart development (Additional file 1: Figure S4). *Cited2* deficiency was found to contribute to tendon aging and degeneration [10], and loss of *Cited2* in mouse ESCs impaired cardiomyocyte differentiation [19] while its overexpression enhanced commitment to the cardiac lineage and differentiation [20]. *Cited2* is also known to regulate trophoblast differentiation [21]. Down-regulation of *Cited2* was associated with high glucose-induced apoptosis in cardiomyocytes in vitro and maternal diabetes-induced ventricular wall thinness genesis [16]. *Cited2* mutations lead to heart disease in humans [22, 23]. Thus, *Cited2* has important role in the development, stem cell differentiation, and functional maintenance of the heart. However, in our study, the differentiation potential of CSCs was not significantly altered by *Cited2* knockdown or overexpression. We previously reported significant changes in CSCs with advancing age, including an increase in their numbers and a decrease in their stemness characteristics [11]. Others have reported that the fundamental properties of stem and progenitor cells such as quiescence, proliferation, self-renewal capacity, and lineage commitment change with age [24]. The proliferation of *Cited2*(−/−) mouse embryonic fibroblasts ceases prematurelythat is associated with a reduction in growth fraction, senescent cellular morphology, and increased expression of the cell proliferation inhibitors [25] .In our study, *Cited2* promoted CSC proliferation and inhibited apoptosis (Figs. 4 and 5). The proliferative capacity of Y-CSCs was higher than that of O-CSCs. Although the results of apoptosis and proliferation in O-CSC indicate a statistically significant, but the differences between the cell populations and apoptosis are very marginal. With advancing age, the fundamental properties of O-CSCs were weaker than Y-CSCs [11]. Due to the different cellular microenvironments, the effects of *Cited2* expression change are also different. With *Cited2* -knockdown on Y-CSCs the cells highly affected by *Cited2*-depletion (Fig. 6) due to apoptotic events, we performed the differentiation tests the impact of *Cited2*-depletion earlier than 3 days (36 h) after transduction. The cell numbers were more than 3 days after transduction, whereas the cells differentiation was still poorly affected by *Cited2* depletion (Additional file 1: Figure S5). Thus, with *Cited2* -knocdown in Y-CSCs decreased proliferation and promoted apoptosis while having little affect on CSC differentiation potential. *Cited2* overexpression promoted CSC escape from quiescence (G0 phase of the cell cycle) and entry into cell division; however, with increased aging, the cells were blocked in G0 phase.

## Conclusions

In conclusion, the results presented here demonstrate that *Cited2* plays an important role in cell cycle progression and in maintaining the balance between CSC proliferation and apoptosis in the process of aging without influencing cell fate decisions. These effects may result either from a direct effect of *Cited2* or as a consequence of the spontaneous differentiation process induced by *Cited2* with aging. Thus, CSCs in aging mice with reduced levels of *Cited2* are compromised in their ability to remain undifferentiated under conditions supporting CSC proliferation and self-renewal. These results provide new insights into the biological role of *Cited2* that may be useful for optimizing cell-based therapies using CSCs.

## Additional files

Additional file 1: **Figure S1.** Differentially expressed genes identified. **Figure S2.** STC (Series Test of Cluster) analysis of differentially expressed genes. **Figure S3.** Profile 15 and functional classification of the profile 15. **Figure S4.** The functional classification of *Cited2*. **Figure S5.** The differentiation tests the impact of *Cited2*-depletion 36 hours after transfection. **Figure S6.** DD-PCR for *Cited2*mRNA expression. (DOCX 1384 kb)

Additional file 2: **Table S1.** The differentially expressed genes in profile15. (XLSX 89 kb)

## Data Availability

All data generated or analysed during this study are included in this published article and its supplementary information files.

## Abbreviations

7-AAD: 7-aminoactinomycin D
CITED2: Cyclic AMP response element binding protein (CREB) binding protein (CBP)/p300-interacting transactivator with ED-rich tail 2
CSCs: Cardiac stem cells
cTn I: Cardiac troponin I
dd PCR: Droplet digital PCR
EGFP: Enhanced green fluorescent protein
ESCs: Embryonic stem cells
O- CSCs: Old - CSCs
RT-PCR: Reverse transcription PCR
STC: Series test of cluster
TSPC: Tendon-derived stem/progenitor cells
VWF: Von Willebrand factor
Y- CSCs: Young – CSCs
α-SMA: α-smooth muscle actin
