# Supplementary material for: Cited2 regulates proliferation and survival in young and old mouse cardiac stem cells
Source: BMC Mol Cell Biol. 2019 Jul 17;20:25. doi: 10.1186/s12860-019-0207-2 (PMC6637580; doi:10.1186/s12860-019-0207-2)
Supplement: Supplementary file 1 — Figure S1. Differentially expressed genes identified. Figure S2. STC (Series Test of Cluster) analysis of differentially expressed genes. Figure S3. Profile 15 and functional classification of the profile 15. Figure S4. The functional classification of Cited2. Figure S5. The differentiation tests the impact of Cited2-depletion 36 hours after transfection. Figure S6. DD-PCR for Cited2mRNA expression. (DOCX 1384 kb) [file 12860_2019_207_MOESM1_ESM.docx]

**Figure S1: Differentially expressed genes identified**. A total of 5506 differentially expressed genes identified via pairwise comparisons between differential aging (2-3 months, 4months and 18months) heart tissues. The tree was based on the log_2_ transformation of the normalized probe signal intensity using hierarchical clustering. Red: up expression genes; Green: down expression genes.


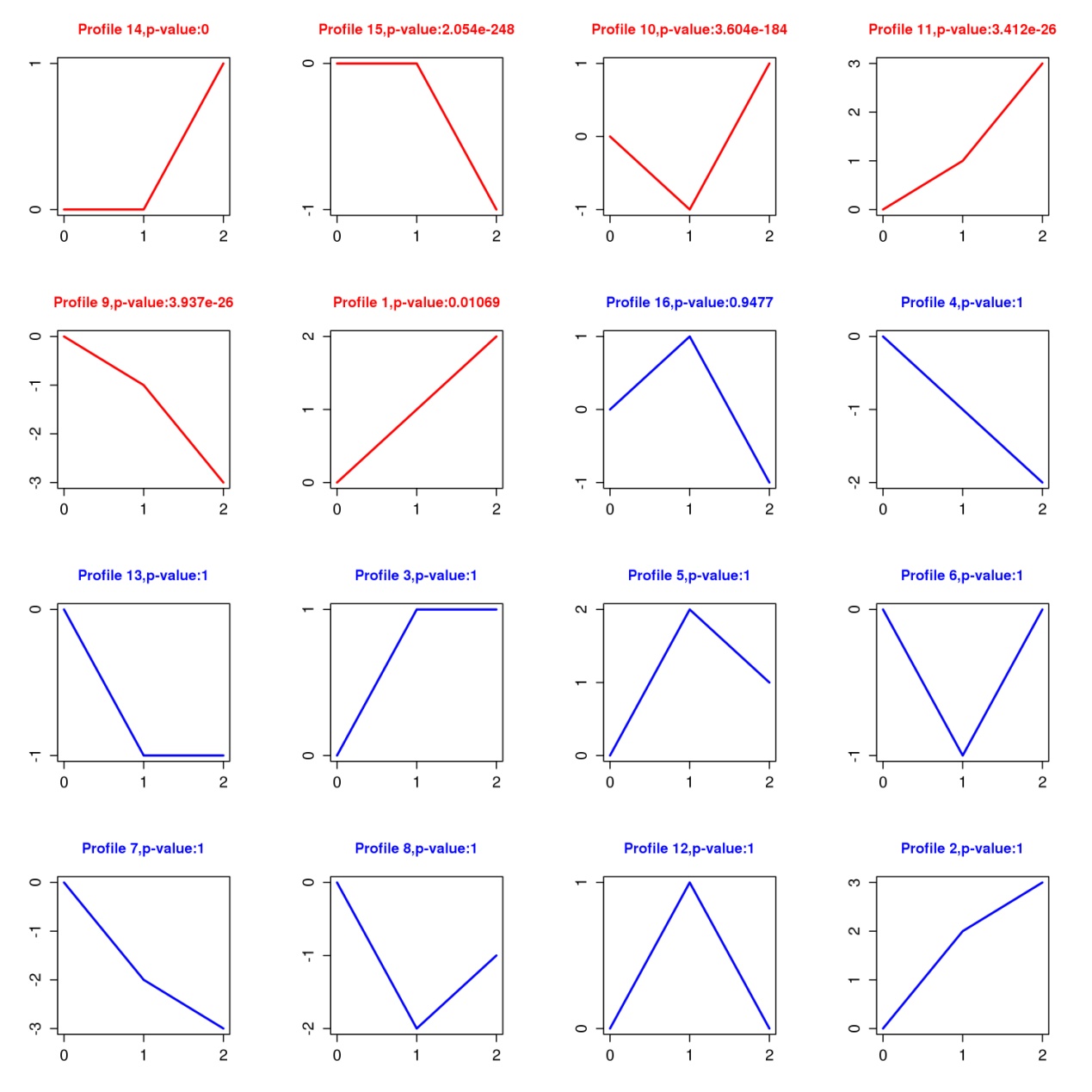


**Figure S2: STC (Series Test of Cluster) analysis of differentially expressed genes.** Dynamic gene expression profiles in 5506 differentially expressed genes are shown 16 clusters. The 16 profiles were ordered on the basis of the *p* value significance of number of genes assigned versus expected. The six red lines are the significant cluster profiles. Y axis indicates the relative gene expression change presented as log2 ratio at the indicated aging points. X axis indicates the aging points (0 represent 2-3 months aging, 1 represent 4months aging and 2 represent 18months aging).


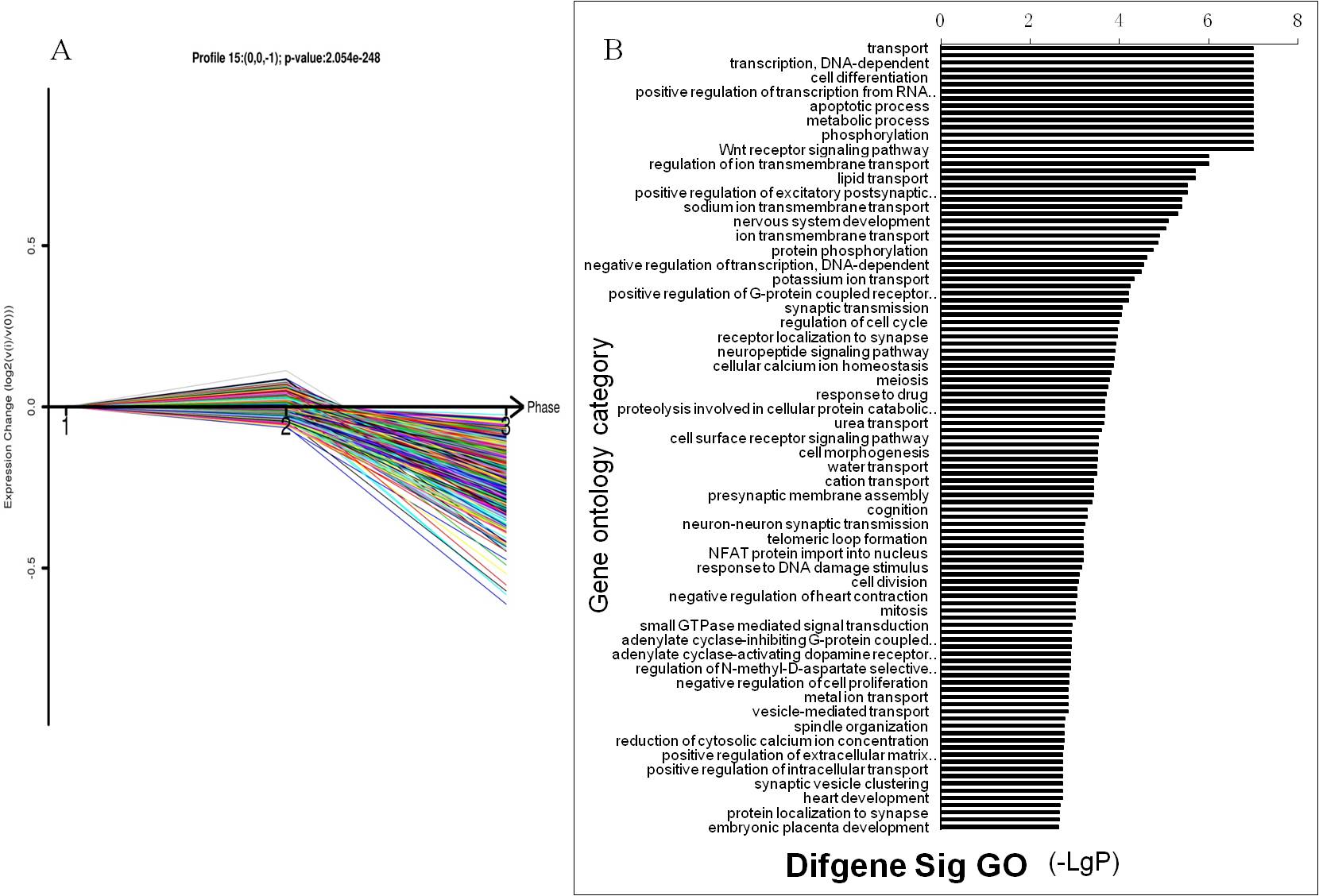


**Figure S3:** **Profile 15 and functional classification of the profile 15.** (A) profile 15 (0,0,-1), 500 genes were expected but 1117 were assigned, p-value = 2.054e-248; (B) Functional classification of the profile 15 was performed according to GO biological processes(0 represent 2-3 months aging, 1 represent 4months aging and 2 represent 18months aging).

**Figure S4:** **The functional classification of *Cited2* in the profile 15.** Functional classification of *Cited2* in the profile 15 was performed according to GO biological processes.


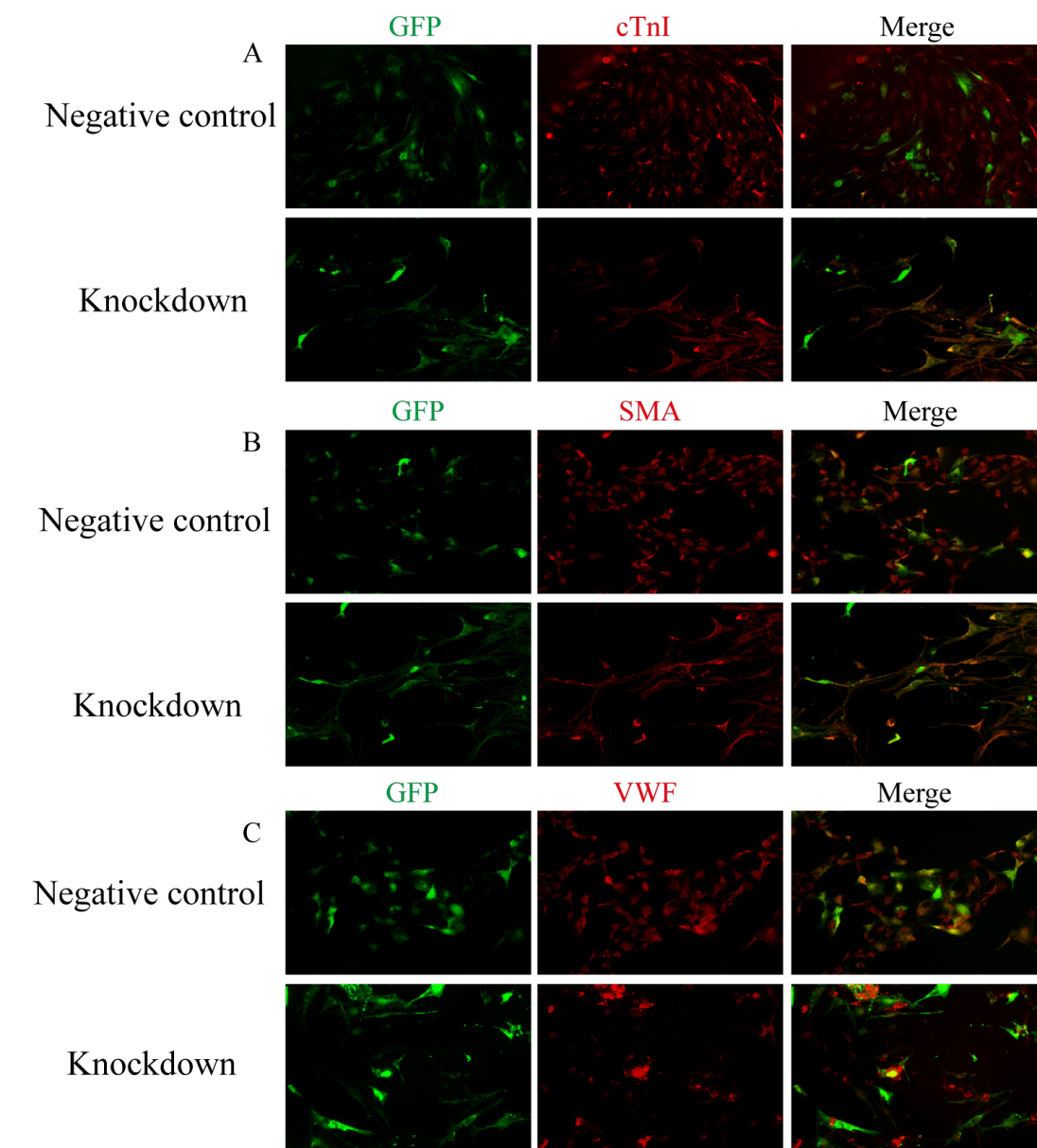


**Figure S5: The differentiation tests the impact of *Cited2*-depletion 36 hours after transfection.**


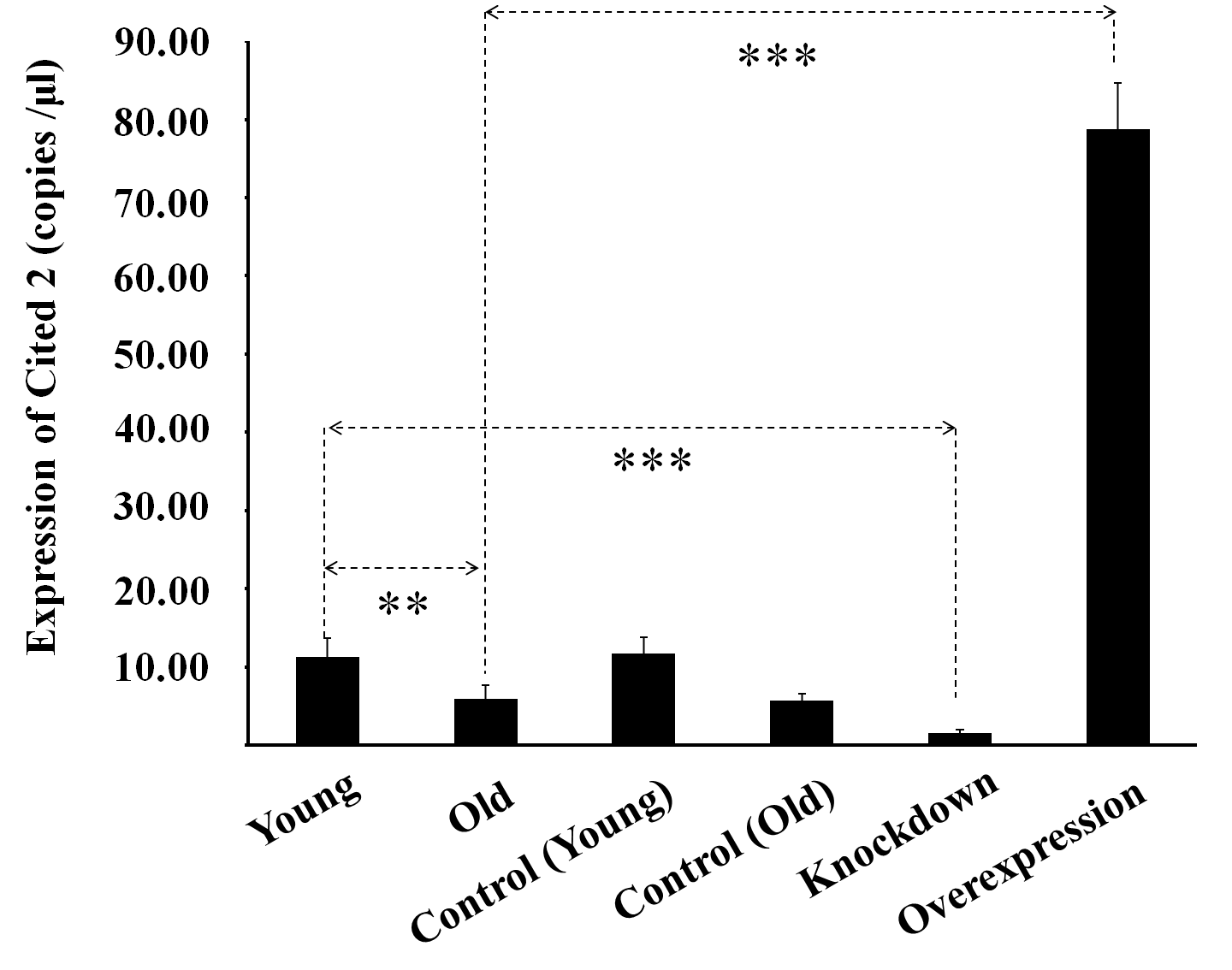


**Figure S6: DD-PCR for *Cited 2* mRNA expression.**

Young is 2-3 months and Old is 20-22 months CSCs. The control was transfected with empty lentivector; Overexpression represented Old CSCs transfected with lentiviral particles of *Cited2*-cDNA；Knockdown represented Young CSCs transfected with lentiviral particles of *Cited2*-shRNA. Data were from three independent experiments and presented as mean ± SD. Statistical analysis was implemented by GraphPad Prism 5, *p < 0.05, ** p < 0.01, and *** p < 0.001.
